# Climate analogs can catalyze cross-regional dialogs for US specialty crop adaptation

**DOI:** 10.1038/s41598-023-35887-x

**Published:** 2023-06-08

**Authors:** Siddharth Chaudhary, Kirti Rajagopalan, Chad E. Kruger, Michael P. Brady, Clyde W. Fraisse, David I Gustafson, Sonia A. Hall, Gerrit Hoogenboom, Rachel L. Melnick, Julian Reyes, Claudio O. Stöckle, Timothy B. Sulser

**Affiliations:** 1 Department of Biological Systems Engineering, Washington State University, Pullman, WA USA; 2 Center for Sustaining Agriculture & Natural Resources, Washington State University, Wenatchee, WA USA; 3 School of Economic Sciences, Washington State University, Pullman, WA USA; 4 Department of Agricultural and Biological Engineering, University of Florida, Gainesville, FL USA; 5 Agriculture & Food Systems Institute, Washington, DC USA; 6 USDA Climate Hubs, Washington, DC USA; 7 International Food Policy Research Institute, Washington, DC USA

**Keywords:** Agroecology, Climate-change impacts

## Abstract

Communication theory suggests that interactive dialog rather than information transmission is necessary for climate change action, especially for complex systems like agriculture. Climate analogs—locations whose current climate is similar to a target location’s future climate—have garnered recent interest as transmitting more relatable information; however, they have unexplored potential in facilitating meaningful dialogs, and whether the way the analogs are developed could make a difference. We developed climate context-specific analogs based on agriculturally-relevant climate metrics for US specialty crop production, and explored their potential for facilitating dialogs on climate adaptation options. Over 80% of US specialty crop counties had acceptable US analogs for the mid-twenty-first century, especially in the West and Northeast which had greater similarities in the crops produced across target-analog pairs. Western counties generally had analogs to the south, and those in other regions had them to the west. A pilot dialog of target-analog pairs showed promise in eliciting actionable adaptation insights, indicating potential value in incorporating analog-driven dialogs more broadly in climate change communication.

## Introduction

Potential climate change impacts on diverse areas such as food production, natural resources, and biodiversity have been well established. However, society continues to fail to take action^1^. One way to address this failure is by presenting more relatable information grounded in past experiences^2^, and climate change analogs have garnered recent attention as a pertinent communication tool^3^. The analog approach takes a “target” location’s future climatic variables and uses statistical distance methods^4^ to identify “analog” locations whose current climatic conditions resemble the target location’s projected future. Thus, these pairings can translate the expectations of an unknown future into known and comparable experiences of peers. Characterizations of analogs have been undertaken for the urban context^3^, agricultural context^5,6^, and general ecological context^4,7^ using raw climatic variables such as seasonal temperature and precipitation. Although communicating more relevant information is a step in the right direction, communication theory emphasizes that transmission^8^ of better information does not on its own result in action^9^, requiring a switch from simple data presentation to an interactive dialog^10,11^. Analogs can be the basis for facilitated dialogs regarding climate adaptation. However, their potential in this aspect is currently underexplored and unrealized, and will likely depend on the application and how the analogs are quantified.

Our objective is to develop acceptable and relevant climate change analogs, using US specialty crop production in the mid-twenty-first century as a case study. We also explore the analogs’ likely utility in facilitating paired target-analog dialogs that could lead to actionable adaptation insights. Specialty crops are legally defined in the US as fruits, vegetables, tree nuts, dried fruits, and other horticultural and nursery crops^12,13^. Within this broad category, we focus on fruits, vegetables, and tree nuts, which encompass a majority (> 90%) of the production acreage. Specialty crops are highly concentrated in California, a state that is increasingly vulnerable to drought and heat stress, but they generate considerable farmgate value in several other states (Table 1). These crops are a useful case study for multiple reasons. First, they are understudied in relation to climate change compared to grain crops such as corn and soybeans^14–16^. Second, they have a larger local economic impact per acre than grains, due to their more complex suite of harvesting, packaging, and processing activities^17^. Third, from a climate change adaptation perspective, specialty crops are challenging because of the long-term investment horizon (for perennial crops), specialized production machinery as compared to grain crops^18^, and large capital and labor investments in production and processing. More broadly, climate change impacts on agriculture are highly nuanced and go beyond the commonly explored impacts on crop yields^16,19^ or land values (i.e., Ricardian analysis)^20^. Multiple climate-impacted dimensions such as pests, diseases, varietal differences, and extreme weather exposure—which have received limited attention—are also critical, and an interactive dialog between a network of specialty crop experts can help navigate the complexities of identifying actionable insights.

**Table 1:** Production area of fruits, vegetables, and tree nuts in the 10 states having the highest farmgate value generated at farms with specialty crops. Source: 2017 USDA AgCensus.

	Production area (M Ha)			Total farmgate value at farms with specialty crops (billions of USD)
	Fruits	Vegetables	Tree nuts	
CA	0.67	0.37	0.82	32.2
WA	0.14	0.13	< 0.01	6.3
FL	0.21	0.09	< 0.01	5.3
OR	0.04	0.06	0.03	2.7
MI	0.05	0.07	< 0.01	2.5
ND	< 0.01	0.03	< 0.01	2.4
ID	< 0.01	0.14	< 0.01	2.4
NC	0.01	0.06	< 0.01	2.0
MN	< 0.01	0.08	< 0.01	2.0
TX	0.01	0.04	0.06	1.7

**Figure 1:**
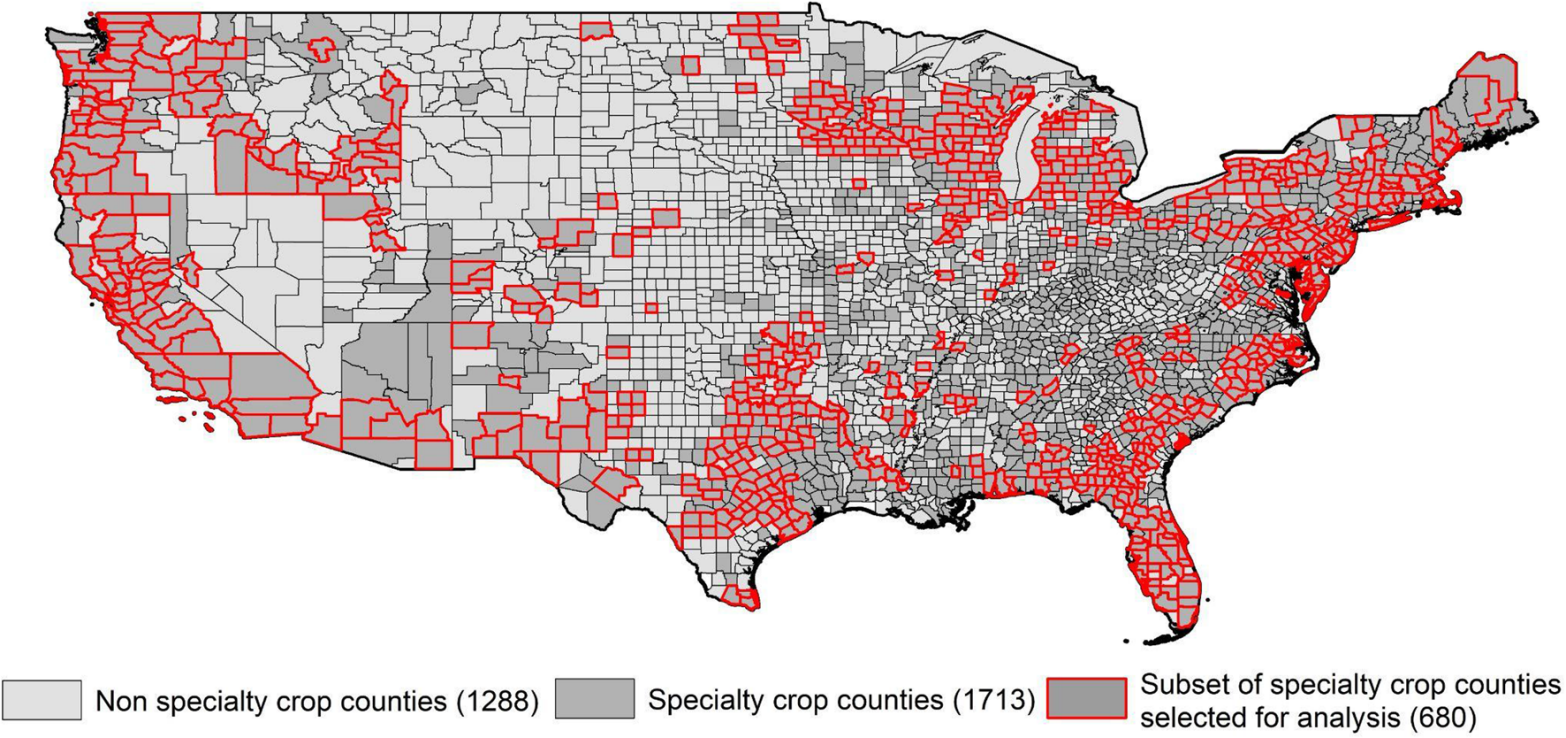
All special crop producing counties in the coterminous US and the subset of 680 counties that account for 99% of the production area.

We simulated analogs for the future climate of 680 target US specialty crop counties that make up 99% of the specialty crop production area (Fig. 1). The county pool for potential analogs includes all 3001 counties in the conterminous Lower 48 states of the US. We focused on a mid-twenty-first century time frame (2040–2070) as it aligns well with the planning and investment horizon of specialty crop growers and is far enough in the future for the climate to be different from current (1990–2020) conditions. Nineteen general circulation models (GCMs), each under two greenhouse gas scenarios—representative concentration pathway (RCP) 4.5 and 8.5—were considered. The future climatic variables in each specialty crop county were compared against the current climatic variables of all US counties to compute a dissimilarity distance metric. We used the sigma dissimilarity metric^4^, as it allowed us to define an interpretable distance threshold to identify a set of "acceptable" analogs, rather than only the closest analog, which may or may not be similar enough for a meaningful dialog.

Beyond being a statistically acceptable analog, selecting variables relevant to the dialog context, and ensuring similarity in the production systems of the target-analog pair can impact the ability of analogs to facilitate meaningful dialog; so we explored these aspects. We first calculated the set of acceptable US county analogs for each specialty crop county based on climate-derived crop-production-relevant variables (context-specific), and compared them to analogs based on seasonal temperature and precipitation variables (generic). Some generic analog data products are available, and this comparison allowed us to determine whether the two approaches identify similar locations as analogs or if generating context-specific analogs helps identify more relevant target-analog pairs. Next, we quantified what fraction of specialty crop counties lack acceptable analogs or have acceptable analogs only in non-specialty crop counties. This is important because dialog and sharing of relevant information would likely be hard—if not unproductive—unless they occur between people familiar with similar production systems. Further, we utilized the 2017 USDA Census of Agriculture statistics to assess similarities in the specialty crops grown in the target and analog counties to obtain a sense of whether the dialogs can focus on insights for managing the same crop in a different environment or if they would primarily relate to opportunities for introducing new crops.

Finally, we piloted such a dialog, convening extension and outreach professionals, including from the US Cooperative Extension System (CES), from target counties in the southeastern US and their analog pairs. The CES is an existing network operated through Land-Grant Universities. It includes specialty crop professionals who work directly with producers of various scales to provide knowledge about the needs and changing conditions faced by the specialty crop supply chain, and thus are well suited to participate in and expand the reach of these interactive dialogs. Other professionals also contribute to translating and disseminating advances to producers, including conservation districts, government agencies, private sector consultants, and non-governmental organizations. Our analysis was performed at the county scale, matching the CES network. These professionals, particularly those in the CES, have long used dialogs within and between regions to facilitate the adoption of new agricultural technologies and practices, although we are not aware of cross-regional dialogs in a climate change context.

## Results

### Importance of developing context-specific analogs

**Figure 2:**
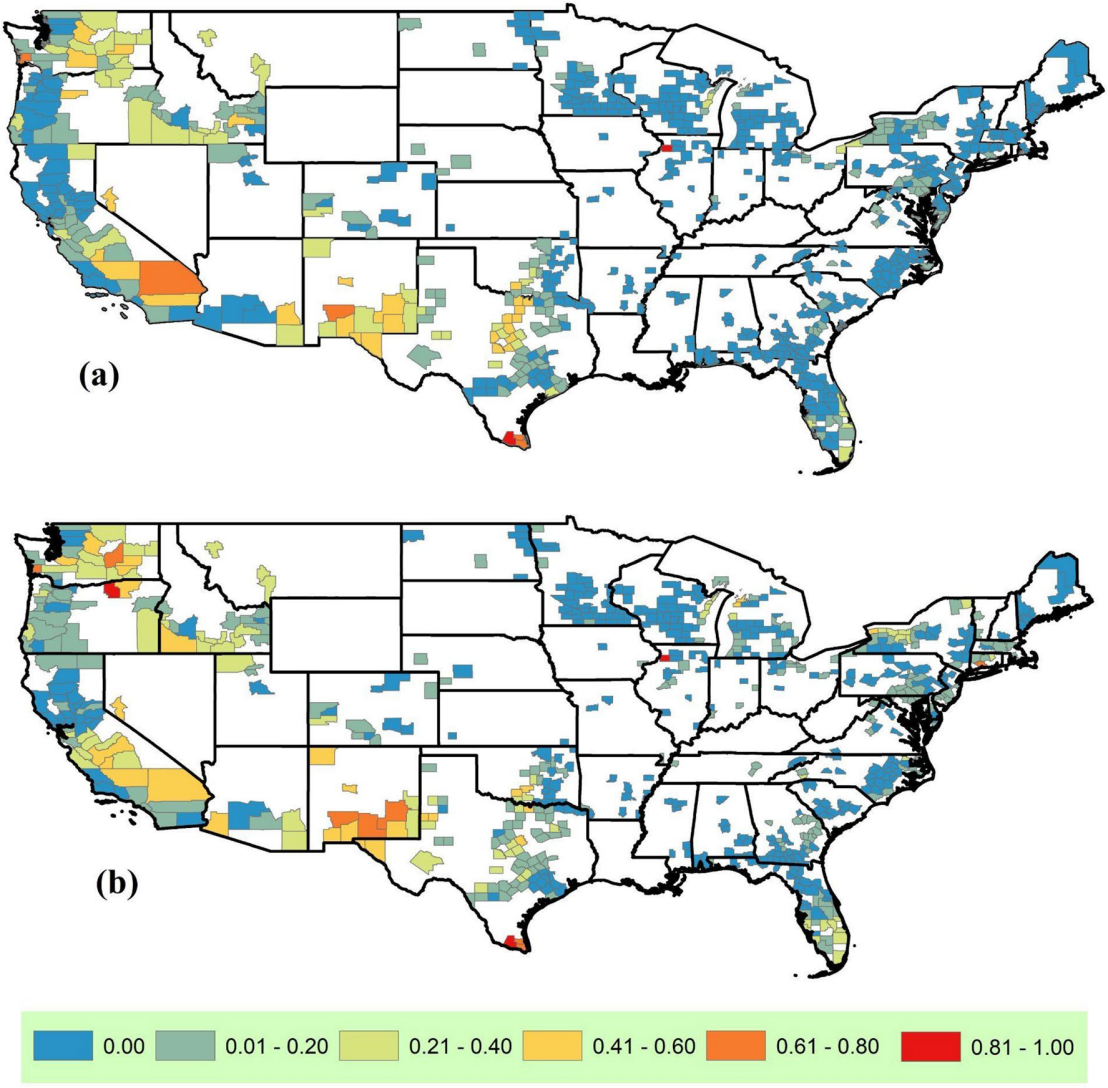
Similarity between the analog set identified using generic vs. context-specific input variables. Each of the 680 specialty crop counties is colored based on the fraction of analog counties that were common to both methods. The similarity ranges from 0.00 when the acceptable analog counties identified using these two sets of input variables are completely different, to 1.00 when the acceptable analog counties are exactly the same in both cases. The analog sets were created using 19 GCMs under RCP 8.5 (**a**) and RCP 4.5 (**b**).

The published literature on climate analogs is nascent and has mainly focused on generic seasonal temperature and precipitation variables (e.g.^3,5–7^). Assuming broad applicability of these generic analogs may not be appropriate for agricultural (and other) applications, given (a) non-linear translations of seasonal temperature and precipitation variables to metrics relevant to a specific context (e.g. growing degree days calculated from temperatures as a metric of plant growth) and (b) different relative weights for categories of input variables. For our case study, we explored the effect of using context-specific input variables. That is, we asked whether using generic raw variables versus crop-production-relevant variables derived from them (growing degree days, heat stress, season length, chill accumulation, and precipitation uniformity) would result in the same set of counties as analogs. While we did not expect the list of acceptable generic and context-specific analog counties to match exactly, surprisingly, the majority of the 680 US specialty crop counties (64% for RCP 8.5 and 50% RCP 4.5) resulted in completely different lists of acceptable analog counties (0% overlap) between the two approaches (Fig. 2). The sensitivity of the selected analog to the input variables (a) emphasizes that identifying analogs based on context-specific variables is likely critical to having target-analog pairs that can result in an effective dialog, and (b) allows us to customize analogs for different dialogs. Moving forward, finding the right balance of context-specificity is a challenge that needs to be addressed by the community via formal evaluations of interactive dialogs. The remainder of our pilot case study takes a first step in exploring whether these particular context-specific analogs are useful for fostering a meaningful dialog around climate adaptation in specialty crops.

### How often do targets have analogs that also grow specialty crops?

**Figure 3:**
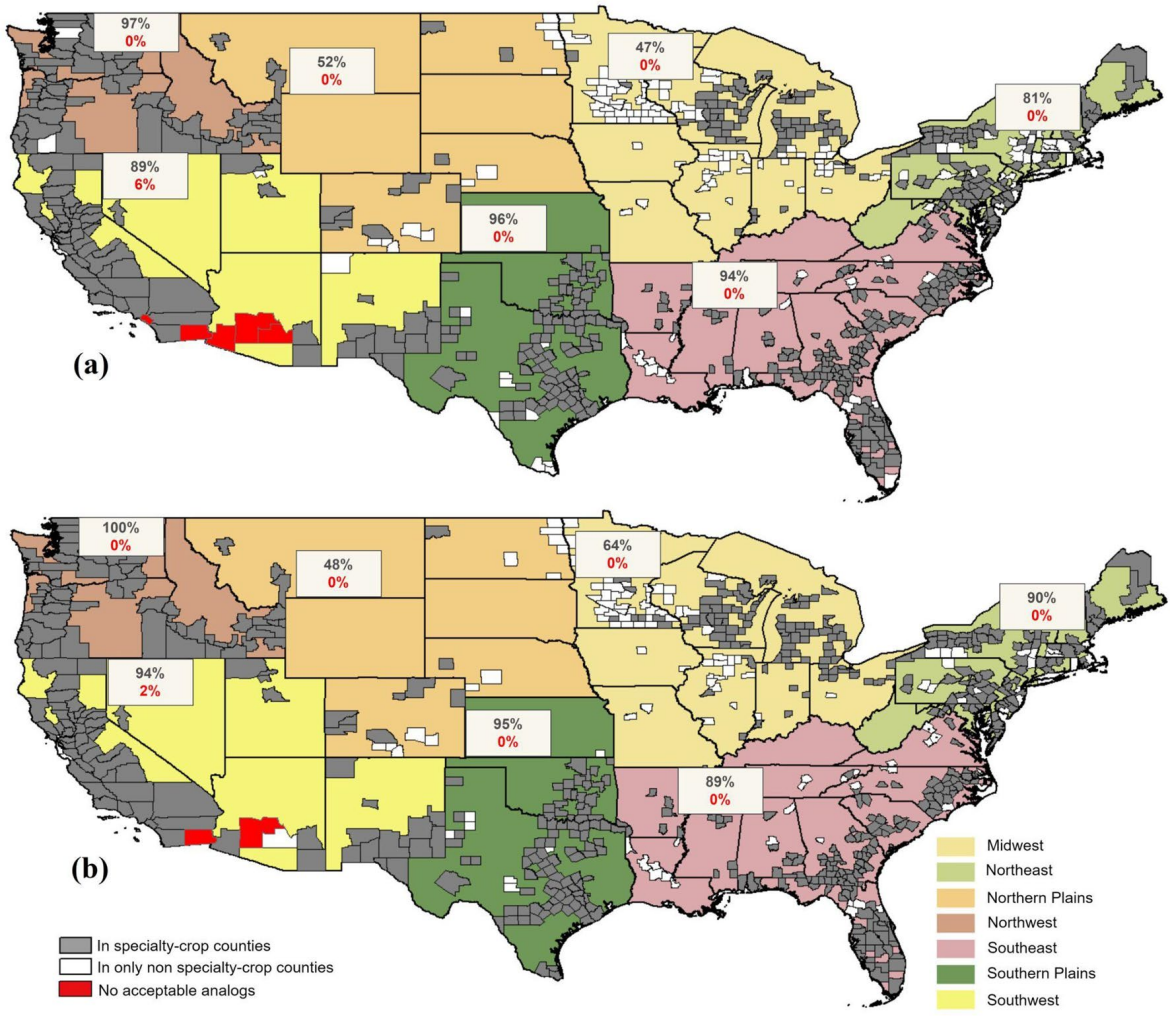
Existence of analog counties with specialty crop production. Each of the 680 specialty crop counties is colored based on whether they have (1) at least one acceptable analog in another specialty crop county (gray shading), (2) acceptable analogs only in non-specialty-crop counties (white shading), and (3) no acceptable analogs within the US (red shading). Each region in the US is annotated with two numbers: the percentage of the specialty crop counties in that region with analogs in other specialty crop counties (top number), and the percentage with no acceptable analogs (bottom number). The analog sets were created using 19 GCMs under RCP 8.5 (**a**) and RCP 4.5 (**b**).

We found that the majority of US specialty crop counties (80% for RCP 8.5 and 84% for RCP 4.5) have some acceptable analogs in other specialty crop counties (Fig. 3). This indicates that US specialty crop production is likely an excellent case study with potential for facilitating dialogs that can inspire change and help sustain future production and related livelihoods. There are regional differences, with the Midwest and Northern Plains having a lower fraction of specialty crop counties with acceptable analogs in other specialty crop counties (Fig. 3). However, these two regions account for < 10% of the national specialty crop production area^21^. The Southwest is a major specialty crop production region that had a handful of target counties without acceptable analogs in the US (6% for RCP 8.5 and 2% for RCP 4.5) (Fig. 3). In fact, their sigma dissimilarity values were high enough for them to be considered novel climates that have not been observed in the US.

### How similar are the specialty crops grown in target-analog pairs?

**Figure 4:**
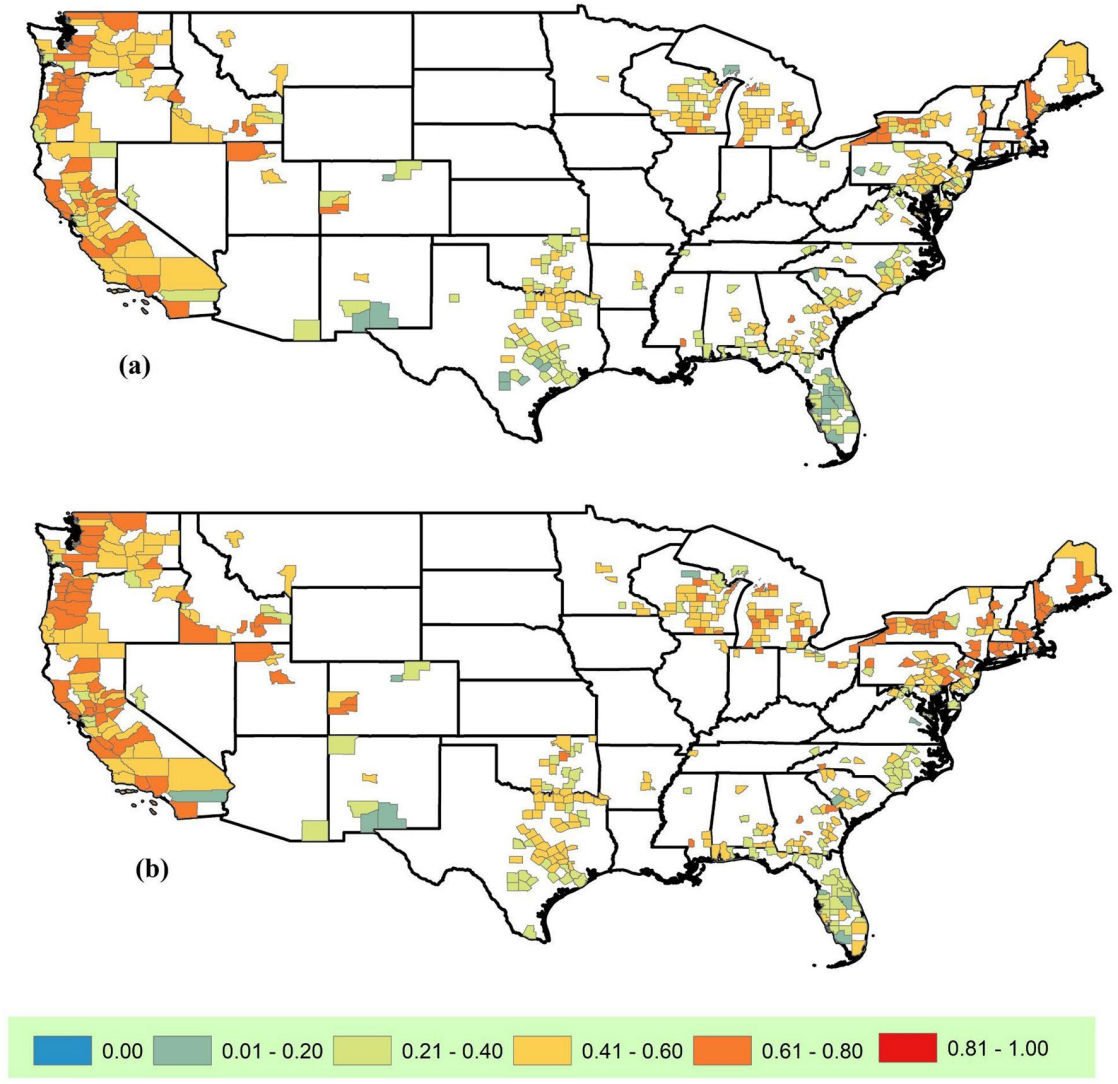
Maximum specialty crop match fraction for each target. This is obtained by comparing the specialty crop mix across each target and acceptable analog pair to calculate a crop match fraction (the fraction of all the crops in the crop mix that occur in both target and analog counties), and then taking the maximum match fraction for each target county. The analog sets were created using 19 GCMs for RCP 8.5 (**a**) and RCP 4.5 (**b**).

There is a wide diversity of specialty crops, so the conversations across target-analog pairs can be around how to manage the same crop for a different future climate (if similar crops are grown) or opportunities for diversifying into new crops. By comparing the list of specialty crops grown (crop mix) that are common to both the target and analog in each pair, calculating the fraction of crops that are common (match fraction) in each pair, and filtering the maximum match fraction for each target county (one target can have multiple acceptable analogs), we found that at least 20% of the specialty crop mix is common for the majority of target-analog pairs (93% of counties under RCP 8.5 and 97% under RCP 4.5). Even aiming for 40% of the specialty crops to match, we have 63% of counties under RCP 8.5 and 73% under RCP 4.5 (Fig. 4). The specialty-crop match fraction is high in the West and Northeast US where this 40% match threshold is met by 85% of the target-analog pairs under RCP 8.5 and 86% under RCP 4.5. Even a match as high as 60% is met by 30% of the target-analog pairs in these regions under RCP 8.5 and 46% under RCP 4.5. This indicates a higher potential for fruitful dialogs around changes in management practices for existing crops in these regions as compared to the Southern Plains and the Southwest US where insights are more likely around opportunities to diversify the crop mix. This analysis was performed based on 556 out of the 680 target counties which had a complete crop list.

### Where are the analogs?

**Figure 5:**
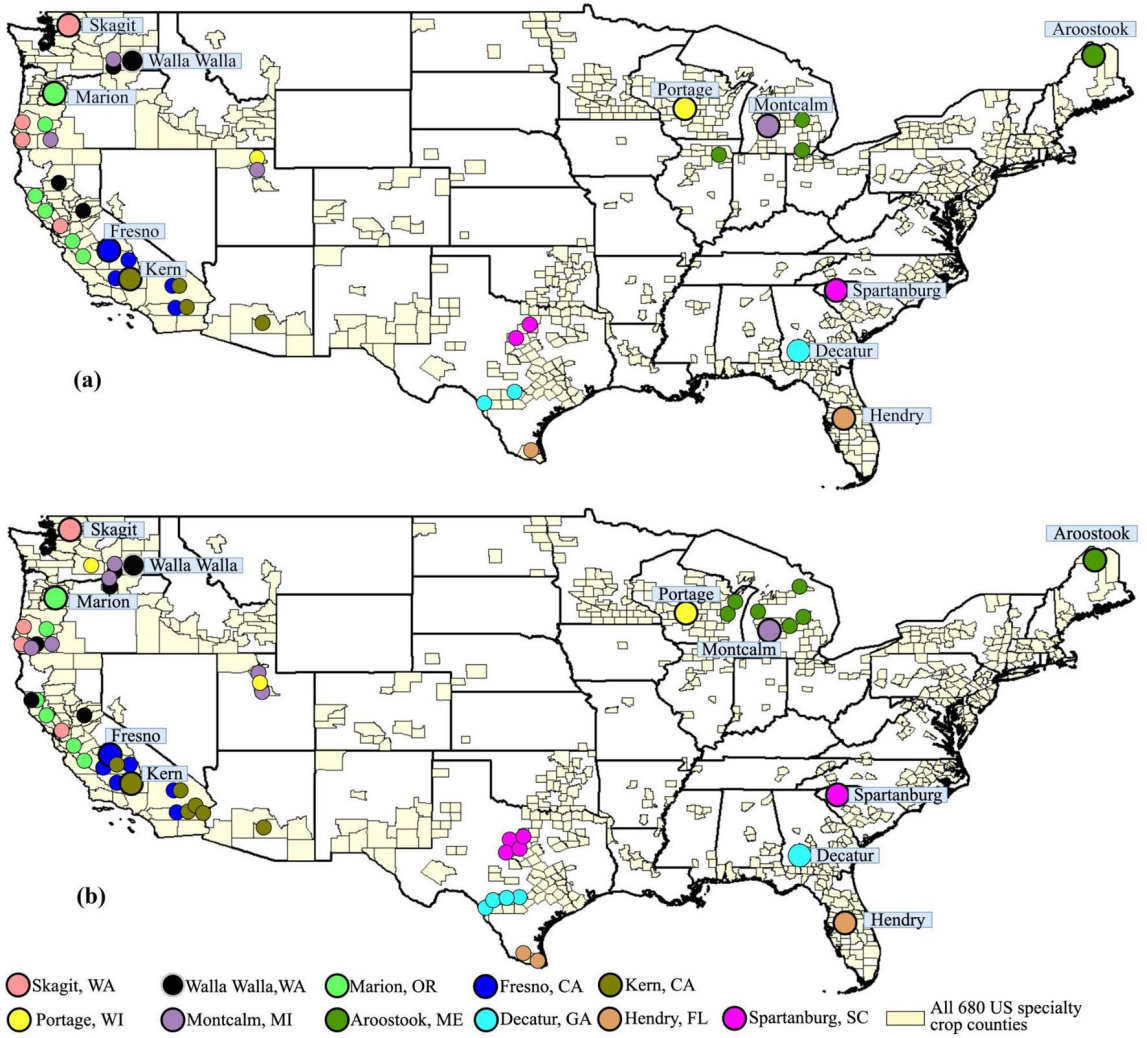
Spatial distribution of all acceptable analogs for select top-producing specialty crop counties for the mid-century (2040–2070) based on 19 GCMs for RCP 8.5 (**a**) and RCP 4.5 (**b**). Target-analog pairs are shaded in a similar color with the target county annotated with the name and having a larger circle size.

The acceptable analogs for counties in the US West generally have analogs further south (Fig. 5). Midwest, Northeast, and Southeast US specialty crop counties generally have analogs in the western direction, sometimes a long geographic distance away. For example, Montcalm County in Michigan has acceptable analogs in parts of southeast Washington. A target county can have multiple acceptable analogs because multiple counties can have similar historical conditions and also because we used 19 GCMs to capture model uncertainty, resulting in different acceptable analogs. Results are generally similar across RCPs 4.5 and 8.5 except for targets in the Northeast. A subset of counties are shown here for visual clarity. An interactive web tool that provides the list of analogs and the crop mix for all 680 target counties is available at https://agclimatechangetools.cahnrs.wsu.edu/ for public use.

### Pilot dialog between Southeast US target counties and their analogs

We piloted a two-phase process to explore analogs’ utility in facilitating paired target-analog dialogs that could lead to actionable adaptation insights. First, in January 2022 we hosted a national-scope orientation webinar for extension professionals where we (a) described the analog approach and shared our analog results and (b) introduced our interest in facilitating target-analog paired dialogs to explore the kinds of discussion and insights that might arise. These extension professionals were invited through a national CES network advertising effort targeting states where the analysis had identified acceptable analogs (Washington, Oregon, California, Wisconsin, Minnesota, Michigan, New York, Maine, North Carolina, South Carolina, Texas, Florida, and Georgia). In some cases, we also directed invitations to specific individuals, based on recommendations from state CES administrators and team members. The orientation webinar (offered twice) included 95 participants from 29 states with specialty crop counties (as well as a few participants with a nationwide focus). During the webinar discussion multiple participants expressed interest in exploring paired target-analog discussions and participants from one target-analog pair were invited to proceed to the second phase of the pilot that involved dialogs.

This second phase centered around a virtual small-group workshop in March 2022. The workshop focused on the Southeast and Southern Plains regions, and involved nine specialty crop extension professionals from target counties in Florida (FL), Georgia (GA), and South Carolina (SC) and similar professionals in their analog counties in eastern Texas (TX) (cyan, fuchsia and brown in Fig. 5). We focused on these states because they had acceptable analog pairs (per our analysis) and had strong participation of extension professionals during the orientation webinar. Participants in the second phase included those who expressed interest at the orientation webinar and others recommended by them. During the workshop we used the following guiding questions to foster discussions among extension professionals working in the target and analog counties: (1) how is climate change impacting specialty crop production in the counties where you work? And (2) what production issues and practices in these regions confer resilience to climate change impacts? In the final portion of the workshop we also asked participants about their perspectives on the usefulness of climate analog pairs as a mechanism to identify and foster connections that lead to a dialog regarding potential adaptation alternatives.

During the workshop discussions three specialty crops arose as the focus: peaches (FL, GA, TX, SC), blueberries (GA, SC, TX), and leafy greens (GA, SC, TX). The team recorded notes of the discussion and collectively interpreted them resulting in a list of four categories of potentially actionable insights (Table 2). Three categories reflected types of actions that growers or other entities (e.g., breeders) could take to foster adaptation. The fourth reflected a transformation in production systems (new opportunities and potential associated challenges category in Table 2). The information in Table 2 was then shared with the participants to confirm accuracy. While we do not know yet if these insights will lead to action in the target counties, they provide interesting examples of what can result from such dialogs, and point to the utility of expanding beyond the pilot and formalizing a process for initiating such dialogs.

**Table 2:** Categories and examples of actionable adaptation insights that arose from the dialog between CES professionals from target counties in the Southeast and their counterparts from analog counties in Texas.

Insight type	Example insights for the target countries from the analogs
Changes to management practices	Pest management may need to transition from the current part-of-the-year time frame to year-around management
Varietal selection	Peach and blueberry producers may need to consider varieties with lower chill requirements
Priorities for breeding programs	Peach and blueberry varieties with a range of chill requirements are already available, yet breeding programs may need to consider the future climate in variety development to ensure producers can meet key market windows that bear a premium
	Breeding for late flowering is an option to address the increasing vulnerability to blueberries to late frost, though there are likely trade-offs with harvest timing and fruit quality with market implications
New opportunities and potential associated challenges	Introduction of pecan production, although the question of what crops could grow in the sandy soils of FL remains
	Introduction of controlled environment agriculture, although extreme weather exposure (e.g., hurricanes) could pose a significant challenge to infrastructure

## Discussion

Communication theory points to the importance of dialog, co-production, and locally-relevant information to translate scientific knowledge to action^22–26^. While it is intuitive and critical to host discussions with a range of stakeholders from within a region which is the historical basis of extension systems^27^, climate analogs appear to be a useful tool to guide pairing with experts in other regions who can translate the unknowns into prior experiences and play an important complementary role in a knowledge-sharing dialog. Though target-analog pairs in the Southeast region produce less similar specialty crops compared to other regions (Fig. 4), the specialty crops that emerged as the focus of discussion and the insights that arose (Table 2) suggest that analogs can be an effective mechanism for identifying and fostering ongoing connections across disparate regions. Dialogs within established social networks have been shown to be a key factor in the adoption of innovation, including around climate adaptation^27,28^. Our pilot suggests there is an opportunity to enable exchange of actionable information across a larger sampling of regions. These paired dialogs could lead to the establishment of new and effective social networks across a larger set of geographical pairings than might otherwise be pursued. Formalizing the structure of the dialog and evaluation of the dialog content would be critical to expanding beyond the pilot proof-of-concept considered in this study.

For the case of specialty crops, two aspects make analogs promising: the high proportion of specialty crop counties with acceptable analogs and the similarity of crop mix between target-analog pairs. This is especially notable for geographically separated regions such as the Western and Northeastern US, pointing to the need for a proactive approach to developing the networking opportunities that are a foundational element of an adaptation dialog. This is particularly important for specialty crops, as they stand in contrast to the major grains (corn, wheat, and soybeans) that typically have contiguous production regions and that are the focus of many of the prominent research articles on climate change adaptation (e.g. Burke et al.^15^, Schlenker et al.^16^). Our pilot process showed early promise for the paired dialog approach to foster action-oriented information exchange between crop experts. The new context-specific target-analog pairs developed in this study can form the basis for facilitating discussions and expanding the network engaging in dialog, which could ultimately contribute to the successful adaptation of these important crop production systems to the challenges of climate change.

The analog calculations in this study did not account for some important environmental factors that affect production, such as soil characteristics and continued irrigation water availability (both of which were raised during the pilot dialog). However, the strength of an interactive dialog format is that it can allow the integration of these unique local considerations into the conversations as well as deliberations on differences and alternative management strategies. Additionally, our analog calculation methods were built on prior work^4^ that calculates distances based on mean climatic variables while accounting for the historical range. A potential future improvement in this approach would be to employ methodologies that compute distances across the entire distributions of the climatic variables or include a time series analysis that addresses the temporal structure of heat waves and precipitation events. Even with the current approach, distributions of variables across target-analog pairs can be compared ex-post, and differences in distribution can be highlighted as discussion points for the dialog on an as-needed basis.

While this paper is solely focused on specialty crops in the US, climate analogs can be relevant in other agricultural systems across the globe to help facilitate paired information exchanges that can catalyze climate change action and adaptation. Climate analogs that incorporate local knowledge on thresholds and nonlinearities related to climate, productivity, and profitability could play an important role in providing key science-based evidence for economic optimization that informs high-value investment, such as planning for new plantings of perennial crops that often exceed USD $50,000/ha. Additionally, this concept can be used to go beyond looking at crop production to addressing needs across the agricultural supply chain, such as in the planning and location of storage facilities, food processing facilities, and transportation hubs that would require additional investment to adapt to new crops. There are also a range of socio-economic factors affecting cropping systems that could potentially be informed by the analog concept, like labor needs, federal policies, and government programs such as crop insurance, disaster assistance, or conservation programs that impact and influence producers’ decision-making.

While extending the analog concept to multiple applications, success likely hinges on identifying acceptable analogs with context-specific metrics derived from climatic variables because the analog set can be quite different from the one based on generic seasonal and temperature variables, as demonstrated in our case study. The target-analog paired network can create the initial stepping stones in an ongoing collaborative dialog that ultimately translates science into action. This is especially important in applications involving working lands where the impacts of climate change are complex, and effective communication models for translating science to action require an interactive dialog rather than simple information transmission.

## Methods

### Meteorological inputs

Historical data were based on the gridMET data product^29^ available at a 1/24th degree (~ 4 km) spatial resolution for the conterminous Lower 48 states of the US for 1979–present with our analysis period for “current” conditions being 1990–2020. Future projections were based on 19 GCMs that were part of the Coupled Model Intercomparison Project (CMIP 5) and bias-corrected and downscaled to a 1/24th degree resolution based on the Modified Multivariate Adaptive Constructed Analog (MACA) methodology^30^. These models include BCC-CSM-1, BCC-CSM-1.1-m, BNU-ESM, CanESM2, CCSM4, CNRM-CM5, CSIRO-Mk3-6-0, GFDL-ESM2G, GFDL-ESM2M, HadGEM2-CC365, HadGEM2-ES365, INMCM4, IPSL-CM5A-LR, IPSL-CM5A-MR, IPSL-CM5B-LR, MIROC5, MIROC-ESM, MIROC-ESM-CHEM, and MRI-CGCM3. Data for the 2040–2070 timeframe for RCPs 4.5 and 8.5 were used. RCP 8.5 is at the 90th percentile of the no-climate-policy scenarios with relatively higher temperature increase projections. RCP 4.5 assumes mitigation of greenhouse gas emissions with relatively lower temperature increases, although these differences are more prominent post our mid-century analysis time frame. Daily maximum and minimum temperature and precipitation variables were used in this study. The 1/24th degree resolution data were re-gridded by linear interpolation to a 1/16th degree resolution for computational efficiency.

### Derived variables and temporal aggregation

We chose the context-specific (specialty-crop-production relevant) variables accounting for key biologically important processes that affect production across all seasons of the year. Growing degree days (GDD) is a predictor of plant growth and development and is a function of heat unit accumulation between the crop-specific base and upper temperature thresholds. It is calculated as described in Miller et al.^31^. One unit of growing degree days is indicative of exposure to 1 °C between the thresholds for 1 day. For our study, we assumed the base temperature of 0 °C (32 °F) and an upper threshold of 29.44 °C (85 °F) and calculated annual GDD accumulation. This cutoff/threshold value is generically applicable to multiple specialty crops. The frost-free season length dictates which crops and varieties can grow and when. Both GDD and the frost-free season length are widely used indicators in crop yield response studies (e.g., Kukal and Irmak^32^). In addition, heat stress, especially during the summer months, can impact crop production^33^. We use heat degree hours—which incorporates both the time and intensity of exposure to damaging temperatures above a certain threshold—as a measure of heat stress. One heat degree hour is equivalent to exposure to temperatures 1 °C above the threshold for 1 h. We used an hourly temperature threshold of 32 °C (89.6°F). We estimated hourly temperatures from daily maximum and minimum temperatures using a sine curve disaggregation^34^ and calculated heat degree hours from this hourly data. For perennial cropping systems, winter dynamics are important, and we used chill hour accumulation as a representative metric. It is a measure of the number of hours of exposure to temperatures between 0 °C (32 °F) and 7.22 °C (45 °F)^35^. This is calculated for the season between 1^st^ October of the prior year and 31st March of the current year as implemented in the chillR package^36^. A lack of sufficient chill accumulation can lead to non-uniform bloom, flower abscission, and reduced fruit set which result in negative production impacts^37^. Finally**,** we were interested in a metric that captures the differences in precipitation patterns between the Western (winter precipitation with dry summers) and Eastern (generally uniform precipitation throughout the year with humid conditions) US. While having multiple monthly precipitation variables is an option, as Williams et al.^38^ note, having a limited number of variables is important for distance metric calculations. Therefore, consistent with the approach taken by the widely used Koppen climate classification^39^, we use the difference in precipitation between the wettest and driest months as our metric. This metric is also correlated with the relative differences in humidity during the growing season (as evident from the Koppen climate classes), which translates to important differences in pest and disease pressures in cropping systems^40^.

The temporal aggregation is summarized in Table 3. The climate analog calculations require three climate variable datasets (a) current climate normal: 30-year mean across 1990–2020 (b) projected future climate normal: 30-year mean across 2040–2070 and (c) annual historical observations across 1990–2020 to calculate the interannual climatic variability. The gridMET dataset^29^ was used to calculate both (a) and (c).

**Table 3:** Summary of derived variables used in our analysis.

Agro-climatic variable	Aggregation	Cutoff/threshold
Growing degree days	Annual^+^	Base temperature of 0 °C (32 °F) and an upper threshold of 29.44 °C (85 °F)
Production season length	Annual^+^	Consecutive frost-free days
Heat degree hours	Seasonal*	32 °C (89.6°F)
Chill hours	Seasonal^#^	Temperature range of 0–7.22 °C (32–45°F)
Precipitation uniformity	Annual*	Not applicable
Temperature	Seasonal*	Not applicable
Precipitation	Seasonal*	Not applicable

^+^Annual aggregation implies a calendar year aggregation.

*Seasonal implies 4 groups of months in a year with 3 months in each group starting from January each year with the exception of chill hours.

^#^The season definition for chill hours is October 1st of the prior year to March 31st of the current year.

### Agricultural grid identification

The climate data were aggregated only for the agricultural grids within a county. These grids were identified based on the 2020 USDA NASS Cropland Data Layer (CDL)^41^ which is an annual georeferenced dataset of cropland use that is created based on satellite imagery analysis and provided at a 30 m resolution. We removed the non-agricultural land use categorization codes (81, 82, 83, 87, 88, 92, 111, 112, 121–124, 131, 141–143, 152, 190, 195) from the CDL dataset to isolate agricultural pixels. The data were then upscaled to the resolution of the climate data input (1/16th degree), by coding a climate data grid as agricultural if at least 1/8th of the grid area comprised agricultural CDL pixels.

### Specialty crop county characterization and crop mix

The 2017 USDA National Agricultural Statistics Service (NASS) Census of Agriculture (NASS Census)^21^ provides multiple county-level agricultural production characteristics including the list of crops produced, and the production area and market value of each crop. The NASS Census data were accessed via the census data query tool^21^ with table numbers 29, 31, and 32 corresponding to vegetables, tree fruits & nuts, and berries respectively. Using the aggregate total statistics for these crop groups, we coded all 3001 counties in the Lower 48 conterminous US as specialty crop or non-specialty-crop counties. Of the 1713 counties that were coded as specialty crop counties we subset 680 counties that made up 99% of the specialty crop acreage for further analysis (Fig. 1). We created an exhaustive set of (a) all crops grown in the county (crop mix) and (b) all focus specialty crops—from the fruit, vegetable, and nut categories—in each county (specialty crop mix). Due to disclosure and missing data issues we were able to create a complete crop list only for 556 out of the 680 counties.

### Climate analog calculation

Two sets of analog calculations were performed. The first is a generic analog based on temperature and precipitation. The second is a context-specific analog based on derived variables: annual growing degree days, frost-free season length, summer heat degree hours, chill hours, and the precipitation difference between the wettest and driest months.

Analogs were calculated following the approach of Mahony et al.^4^ which (a) adapts the standardized Euclidean distance into a Mahalanobis distance with variables scaled by their interannual variability and (b) interprets distances as *sigma dissimilarity* percentiles in a chi distribution. The advantages of this approach are that it addresses issues arising from variable values having different scales and variance inflation arising from correlations across variables. Additionally, it addresses the impact of dimensionality on distance calculations—which hinders the ability to compare distances—by translating distances into a chi distribution space. The one distinction in our approach was that we calculated interannual variability in climatic variables from the gridded data product from which climate normals were calculated as opposed to matching grids with the nearest weather station data. Sigma dissimilarity was calculated for 680 specialty crop counties by comparing the future climate of each of these counties with the historical climates of all 3001 counties in the conterminous US for 19 GCMs.

A sigma dissimilarity of two (2σ) represents the 95th percentile of the chi distribution, while 4σ represents the 99.994th percentile. Counties with a sigma dissimilarity distance ≤ 2σ are considered acceptable analogs while those with a sigma dissimilarity distance ≥ 4σ are considered novel climates. For any target county, acceptable analogs may be either specialty crop or non-specialty-crop counties and we make a distinction between the two. The set of acceptable analogs for each target county is first identified for each GCM. Then, to create an overall set of acceptable analogs across all GCMs, we filter only counties that resulted as an acceptable analog in at least five GCMs (which is equivalent to analogs common across > 25% of the GCMs). While the threshold is arbitrary, the purpose was to constrain the acceptable analog set to counties common across multiple models rather than outliers. We did perform a sensitivity analysis on the threshold (for values of 3, 5, 7, and 10 GCMs). The estimated percentage of specialty crop counties with analogs in other specialty crop counties did not vary by more than 10% with the different thresholds, nor did the threshold affect the interpretation of results.

### Simulation pipeline

Meteorological and derived inputs were first temporally aggregated to a monthly timestep for each grid and then spatially aggregated for each of the 3001 counties in the Lower 48 conterminous US by averaging across all the agricultural grids in each county. Then, 680 out of 1713 counties with some specialty crop production were selected as target counties for the analog calculations. This was done by sorting the counties by specialty crop production area and selecting the top counties that accounted for 99% of the acreage. Sigma dissimilarity distance statistics were computed for each of the 680 target counties for 19 GCMs under RCPs 4.5 and 8.5 scenarios. This was done by computing the distance between each mid-twenty-first century future realization for the target county and the historical climate of each of the 3001 US counties for a total of 77.5 million comparisons (680 target counties × 19 GCMs × 2 RCPs × 3001 counties). The distance statistics were used to filter acceptable analogs for each county-GCM-RCP combination, such that the sigma dissimilarity metric is ≤ 2σ. Then the acceptable analog set was finalized for each county-RCP combination by filtering acceptable analogs that are common across at least five GCMs as noted in the prior section.

Acceptable analogs were calculated for two sets of variables (generic and context-specific) and the match fraction was calculated as the ratio of the number of common unique acceptable analogs and the total number of unique acceptable analogs across the union of acceptable analogs from both sets of variables. The acceptable analog set is then grouped into specialty crop and non-specialty-crop analogs to compute the fraction of total acceptable analogs that are specialty crop counties. Finally, the ratio of common specialty crops to total specialty crops for each target county is calculated as follows. For each acceptable analog, we calculated the total count of unique specialty crops from the union of the target and analog specialty crop mix sets. Then we calculated the count of common unique crops and divided it by the total count to estimate the common crop ratio for each acceptable analog. The highest ratio across all acceptable analogs is reported for each target. This last step was possible only for the 556 counties that had complete crop lists.

## Data Availability

The scripts for the analog calculation (a modified version of the code shared by^4^), post-processing scripts, and a csv file of distance metrics and target analogs pairs are available at the GitHub link: https://github.com/agroecosystemsmodelingwsu/AgClimateAnalog. Analogs and the crop mixes for the 680 top specialty crop counties in the US can be visualized at https://agclimatechangetools.cahnrs.wsu.edu/. The raw climate inputs can be accessed at: https://www.climatologylab.org/maca.html.
